# Neuroglial Involvement in Abnormal Glutamate Transport in the Cochlear Nuclei of the *Igf1*^−/−^ Mouse

**DOI:** 10.3389/fncel.2019.00067

**Published:** 2019-03-01

**Authors:** Veronica Fuentes-Santamaría, Juan C. Alvarado, Lourdes Rodríguez-de la Rosa, José M. Juiz, Isabel Varela-Nieto

**Affiliations:** ^1^Instituto de Investigación en Discapacidades Neurológicas (IDINE), Facultad de Medicina, Universidad de Castilla-La Mancha, Albacete, Spain; ^2^Grupo de Neurobiología de la Audición, Instituto de Investigaciones Biomédicas Alberto Sols, Consejo Superior de Investigaciones Científicas-Universidad Autónoma de Madrid, Madrid, Spain; ^3^Centro de Investigación Biomédica en Red de Enfermedades Raras (CIBERER), CIBER MP, Instituto de Salud Carlos III, Madrid, Spain; ^4^Instituto de Investigación Sanitaria del Hospital Universitario La Paz (IdiPAZ), Madrid, Spain

**Keywords:** hearing loss, cochlear nucleus, IGF-1, glutamate receptors, astrocytes, microglia

## Abstract

Insulin-like growth factor 1 (IGF-1) is a powerful regulator of synaptic activity and a deficit in this protein has a profound impact on neurotransmission, mostly on excitatory synapses in both the developing and mature auditory system. Adult *Igf1*^−/−^ mice are animal models for the study of human syndromic deafness; they show altered cochlear projection patterns into abnormally developed auditory neurons along with impaired glutamate uptake in the cochlear nuclei, phenomena that probably reflect disruptions in neuronal circuits. To determine the cellular mechanisms that might be involved in regulating excitatory synaptic plasticity in 4-month-old *Igf1*^−/−^ mice, modifications to neuroglia, astroglial glutamate transporters (GLTs) and metabotropic glutamate receptors (mGluRs) were assessed in the cochlear nuclei. The *Igf1*^−/−^ mice show significant decreases in IBA1 (an ionized calcium-binding adapter) and glial fibrillary acidic protein (GFAP) mRNA expression and protein accumulation, as well as dampened mGluR expression in conjunction with enhanced glutamate transporter 1 (GLT1) expression. By contrast, no differences were observed in the expression of glutamate aspartate transporter (GLAST) between these *Igf1*^−/−^ mice and their heterozygous or wildtype littermates. These observations suggest that congenital IGF-1 deficiency may lead to alterations in microglia and astrocytes, an upregulation of GLT1, and the downregulation of groups I, II and III mGluRs. Understanding the molecular, biochemical and morphological mechanisms underlying neuronal plasticity in a mouse model of hearing deficits will give us insight into new therapeutic strategies that could help to maintain or even improve residual hearing when human deafness is related to IGF-1 deficiency.

## Introduction

Gene knockout studies in mice have demonstrated that insulin-like growth factor 1 (IGF-1), a growth promoting hormone, is essential for the proper functioning of the inner ear (Magariños et al., 2012; Varela-Nieto et al., 2013; Yamahara et al., 2015). A loss of this factor causes significant anomalies in the development of cochlear circuits during postnatal development, which impairs peripheral function in these animals (Camarero et al., 2001, 2002; Cediel et al., 2006; Riquelme et al., 2010). Indeed, immature patterns of cochlear innervation persist in postnatal day 20 (P20) knockout mice and the spiral ganglion is abnormal, phenomena that are associated with increased auditory thresholds between the ages of 1–12 months and that ultimately result in severe sensorineural deafness (Murillo-Cuesta et al., 2011). Consequently, there is hypotrophy and hypoplasia in the cochlear nucleus of adult *Igf1*^−/−^ mice, which develops in parallel with higher wave II amplitudes in auditory brainstem responses (ABRs). The lack of IGF-1 also modifies excitatory but not inhibitory synapses in the cochlear nuclei, evident as an upregulation in the vesicular glutamate-transporter 1 (VGluT1; Fuentes-Santamaría et al., 2016). This modulation involves myocyte enhancer factor-2 (MEF2) transcription factors, which are downregulated in the IGF-1-deficient cochlea and cochlear nucleus, and that play a pivotal role in regulating excitatory synapses (Flavell et al., 2006; Ruffle et al., 2006; Sanchez-Calderon et al., 2010; Rashid et al., 2014).

Glia are closely associated with synapses and they are key regulators of synaptic neurotransmission in the central nervous system (CNS; Bacci et al., 1999; Hansson and Rönnbäck, 2003; Ullian et al., 2004; Slezak et al., 2006). In the healthy brain, quiescent microglial cells are very dynamic, and they act as sensors to maintain environmental homeostasis in the CNS. Along with their well-established role as modulators of brain inflammation, responsive microglia monitor neuronal excitability and they regulate synapse formation, maturation and pruning by secreting factors that affect synaptic responses (Eroglu and Barres, 2010). Under pathological conditions, these non-neuronal cells also represent the first line of defense in the CNS, responding quickly to signals released by injured neurons and by synapses, by modifying their structural appearance and physiology (Wolf et al., 2017). Studies based on glia-free culture of isolated cortex, retinal ganglion cells or spinal motor neurons have revealed that astrocytes also contribute to the formation, maturation and stability of synapses (Nakanishi et al., 1994; Pfrieger and Barres, 1996; Ullian et al., 2001). When extracellular glutamate levels increase, accumulation of this neurotransmitter is avoided by the clearance of glutamate from the synaptic cleft by excitatory amino acid transporters (EAATs) on astrocytes, preventing excess receptor activation and hence, glutamate excitotoxicity (Danbolt, 2001). These non-neuronal cells express both the EAAT1/glutamate aspartate transporter (GLAST) and EAAT2/GLT1 human/rodent carrier subtypes, and although their expression is region-selective (Lehre et al., 1995), GLT1 appears to be responsible for most of the glutamate uptake at excitatory synapses (Rothstein, 1996; Lehre and Danbolt, 1998). More specifically, GLAST and not GLT1 is expressed strongly by inner supporting cells in the developing and adult mouse cochlea (Furness and Lehre, 1997; Furness and Lawton, 2003; Jin et al., 2011), and it is responsible for glutamate removal at the synapse between inner hair cells and Type I spiral ganglion neurons (Glowatzki et al., 2006). Fibrocytes in the spiral ligament and spiral limbus also express high levels of GLAST, suggesting that these cells also regulate cochlear glutamate uptake (Furness et al., 2009). The fact that GLAST-deficient mice accumulate glutamate in the perilymph in response to acoustic overstimulation led to the proposal that this carrier protects against noise-induced hearing loss (Hakuba et al., 2000; Chen et al., 2010). Ultrastructural studies in the mouse have also provided evidence that both Na^+^-dependent glutamate transporters (GLTs) are expressed strongly in the cochlear nucleus, where they might modulate synaptic function (Josephson and Morest, 2003). GLT1 is also expressed by primary auditory neurons and glial cells in the spiral ganglion (Rebillard et al., 2003).

By competing for neurotransmitter binding and/or uptake, GLTs also modify the activation state of receptor populations at nearby synapses (Huang et al., 2004; Galik et al., 2008; Benediktsson et al., 2012). As such, electrophysiological recordings in acute hippocampal slices demonstrate that selective pharmacologic inhibition of astrocyte GLTs modifies the access of mGluRs to glutamate released at interneuron synapses (Huang et al., 2004; Nicoletti et al., 2011). These mGluRs are G-protein coupled receptors (GCPRs) that activate multiple signaling pathways to drive slow neuronal excitation in the brain (Niswender and Conn, 2010). Based on their specific properties, these receptors have been divided into groups I (mGluR1 and mGluR5), II (mGluR2 and mGluR3) and III (mGluR4, mGluR6, mGluR7, mGluR8), each of which seems to be involved in modulating cell excitability and synaptic transmission (Ferraguti and Shigemoto, 2006). The expression of mGluRs in the mammalian and avian auditory system has been seen to vary depending on the specific nucleus and the cell types they contain (Petralia et al., 2000; Lu, 2014). For example, mGluR1 and mGluR2 mRNA and protein has been detected in both the dorsal and ventral subdivisions of the cochlear nuclei, most intensely in the dorsal cochlear nucleus (DCN) where they seem to be located both pre- and post-synaptically (Bilak and Morest, 1998; Petralia et al., 2000; Kemmer and Vater, 2001; Nicholas and Hyson, 2004; Diaz et al., 2009; Martinez-Galan et al., 2010; Carzoli and Hyson, 2011, 2014). However, there is some controversy regarding the expression of the mGluR4 and mGluR7 subunits, and little information is available about mGluR3, mGluR5, mGluR6 and mGluR8 (Lu, 2014).

In the present study, we have assessed whether IGF-1-dependent alterations in synaptic neurotransmission in the cochlear nuclei involve glial cell dysfunction. Given that neuroglia actively participates in regulating synaptic transmission, the expression of ionized calcium-binding adaptor 1 (IBA1) and glial fibrillary acidic protein (GFAP) mRNA and protein was evaluated in mice lacking the *Igf1* gene as markers of microglial and astrocytes, respectively. In addition, as the activity of the glutamatergic system is impaired in *Igf1*^−/−^ mice, possible abnormalities in the expression of the astroglial glutamate carriers (GLT1 and GLAST) and metabotropic glutamate receptors (mGluR1–5) were also investigated.

## Materials and Methods

### Mouse Handling and Genotyping

Mice heterozygous for the *Igf1* gene (*Igf1*^+/−^) were maintained on a hybrid MF1 and 129/sv genetic background to increase the survival of *Igf1*^−/−^ animals (Liu et al., 1993). *Igf1*^−/−^ mice mortality before adulthood is high, although between 20%–30% survived. Male and female *Igf1*^−/−^, *Igf1*^+/−^ and *Igf1*^+/+^ mice littermates were used in this study, aged 4 months (*n* = 24), and they were genotyped as described previously (Sanchez-Calderon et al., 2010). The mice were fed tap water and a standard diet, and they were housed following recommendations of the Federation of European Laboratory Animal Science Associations. Animal experimentation was carried out in accordance with Spanish and European legislation (RD 53/2013; EU directive 2010/63/EU), and the protocols were approved by the Animal Care and Use Committees of Spanish National Research Council (CSIC).

### Cytoarchitecture of the Mouse Cochlear Nuclei

The nomenclature used to define the cochlear nucleus complex was based on previous studies in the mouse (Mugnaini et al., 1980; Martin and Rickets, 1981; Lambert and Schwartz, 1982; Webster and Trune, 1982). The criteria for the classification of cochlear nucleus neurons were based on cell size, shape and location within the nucleus. As in other mammals, the cochlear nuclei can be divided into two major regions termed the DCN and the ventral cochlear nucleus. *The DCN* is a laminated structure composed of three layers; the superficial molecular layer (ml, layer 1), the granule/fusiform cell layer (grl, layer 2) and the deep layer or central region (cr, layer 3) of the nucleus. *The layer 1* is composed of small stellate cells, cartwheel cells, granule cell axons and fusiform cell dendrites. *The layer 2* is made up of diverse neuron types including fusiform cells, also known as pyramidal or principal cells, many granule cells and cartwheel cells while *the layer 3* contains mostly giant cells and tuberculoventral cells. *The ventral cochlear nucleus* is further divided by the cochlear nerve root into posteroventral cochlear nucleus (PVCN) and anteroventral cochlear nucleus (AVCN) subdivisions. According to their size and morphology, there are three main types of ventral cochlear nucleus neurons: (1) globular and spherical bushy cells are mainly located in the AVCN; (2) multipolar cells are present in both the AVCN and the PVCN; and (3) octopus cells are found exclusively in the PVCN. *The cochlear* *granule cell domain* is a continuous sheet of tightly packed granule cells which covers the dorsal and ventral regions of the ventral cochlear nucleus and the granule/fusiform layer of the DCN. A lamina of these cells also extends medially and ventrally to separate the DCN from the PVCN.

### RNA Isolation and Reverse Transcription Quantitative PCR

Mice were sacrificed by barbiturate overdose (Dolethal^®^, 40–90 mg/kg i/p), decapitated and a midline incision was made in the skin of the head to flip it over the eyes and free the skull. Next, a frontal bone cross-section in the skull in front of the olfactory bulbs and a caudal cross-section by the interparietal bone were made. Both sections were joined by a longitudinal section along the sagittal suture, which facilitated partial removal of the frontal and parietal bones and exposure of the encephalon. To locate the cochlear nuclei, different anatomical landmarks including the cerebellum, the inferior cerebellar peduncle and the spinal trigeminal tract were used as reference points (Franklin and Paxinos, 2013). After removal of the overlying cerebellar flocculus, the dorsal and ventral cochlear nuclei of *Igf1^+/+^*, *Igf1*^+/−^ and *Igf1*^−/−^ mice (at least *n* = 3 per genotype) were dissected off the dorsolateral part of the brainstem (Ryugo and Willard, 1985; Juiz et al., 2000; Fuentes-Santamaria et al., 2005; Caminos et al., 2015). Tissue samples were conveniently stabilized using *RNAlater*^®^ and disrupted with a* TissueLyser* system. Total RNA was isolated and genomic DNA efficiently removed using the *RNeasy Plus Mini Kit*^®^ automated on a QIAcube (QIAGEN), according to manufacturer’s instructions. Quality and quantity of RNA was further assessed by using the Agilent 2100 bioanalyzer, only samples with a RIN > 8.5 were used. Using equal quantities of this RNA from each individual mouse as a template, cDNAs were generated by reverse transcription (RT: *High-Capacity cDNA Reverse Transcription Kit*; Thermo Fisher Scientific) and amplified by quantitative PCR (qPCR) in a 7900HT System (ThermoFisher Scientific) as described previously (Fuentes-Santamaría et al., 2016). TaqMan^®^ Gene Expression Assays^1^ (Life Technologies) were used to detect *Iba1* (Mm00479862_g1), *Gfap* (Mm01253033_m1), *Glast* (Mm00600697_m1), *Glt1* (Mm00441457_m1), *mGluR1* (Mm01187089_m1), *mGluR2* (Mm01235831_m1), *mGluR4* (Mm01306128_m1) and* mGluR7* (Mm01189424_m1). All probes used span exon/exon boundaries with the exception of *Iba1* (Mm00479862_g1). Randomly, control reactions were carried out to secure the quality of the reagents used (no cDNA) and purity of RNA preparations (no RT). Hypoxanthine phosphoribosyltransferase 1 (*Hprt1*) and ribosomal protein lateral stalk subunit P0 (*Rplp0*) were used as endogenous reference genes for normalization, and the relative quantity (RQ) was calculated against calibrator samples, as determined by the 2^−ΔΔCt^ method (Livak and Schmittgen, 2001). The data are presented as the mean RQ.

### Characterization of Primary Antibodies

Information about the primary antibodies used in this study is summarized in Table 1. The* anti- NeuN* antibody was produced from the nuclei of mouse brain cells and as described previously (Mullen et al., 1992; Lind et al., 2005), it detects a single 48 kDa band in western blots of mouse brain tissue. It has been used as a neuronal maker as it recognizes the neuron-specific NeuN protein, which is widely expressed in peripheral and central neurons (Rasmussen et al., 2007). The staining pattern described here for the cochlear nuclei matches previous descriptions (Fuentes-Santamaría et al., 2012). The* anti-calretinin (CR)* antibody was raised against the human CR protein and its specificity has been assessed in Western blots of membrane fractions from the cochlear nucleus, recognizing a single specific 31 kDa band (Fuentes-Santamaria et al., 2005). The* IBA1* antibody was raised against a synthetic peptide corresponding to the C-terminal fragment of rat protein, N-PTGPPAKKAISELP-C (Imai et al., 1996). This antibody recognizes a single band with an estimated molecular weight of 17 kDa, and it stains microglia and macrophages in the peripheral and central auditory system (Ito et al., 1998; Sasaki et al., 2001; Fuentes-Santamaría et al., 2012). The *GFAP* antibody was raised against GFAP from bovine spinal cord and it recognizes a single 50 kDa band in western blots that corresponds to the GFAP protein (Debus et al., 1983). GFAP is an intermediate filament protein and it mainly stains astrocytes in the mature CNS. It is important to note that GFAP is a reliable marker to label reactive protoplasmic and fibrous astrocytes but since some resting astrocytes express this marker only weakly, they might not be detected by immunohistochemistry (Sofroniew and Vinters, 2010). GFAP staining in the mouse cochlear nuclei with this antibody was consistent with previous studies in rodents (Fuentes-Santamaría et al., 2017). The* anti-EAAT1 (GLAST)* antibody was raised against a peptide mapping to the C-terminus of human EAAT1 and it detects a single band at approximately 65 kDa in Western blots of rat brain tissue. EAAT1 is an excitatory amino acid transporter expressed mainly in astrocytes and staining of the mouse cochlear nucleus with this antibody matched previous observations (Furuta et al., 1997; Schmitt et al., 1997). The* anti-EAAT2 (GLT1)* antibody was raised against a synthetic peptide from the carboxy-terminus of rat GLT1 and its target is also an excitatory amino acid transporter mainly located in astrocytes. Its specificity and staining pattern have been described elsewhere (Furuta et al., 1997; Atoji and Islam, 2009).

**Table 1:** List of primary antibodies.

Primary antibody	Immunogen	Host	Code/clone	Dilution	Manufacturer
NeuN	Recombinant mouse NeuN	Guinea Pig	ABN90	1:2,000	Millipore, Billerica, MA, USA
CR	Recombinant human CR	Rabbit	7699/3H	1:1,500	Swant, Bellinzona, Switzerland
Iba-1	C-terminus of Iba1′ (N′-PTGPPAKKAISELP-C′)	Rabbit	019-19741	1:2,000	Wako Pure Chemical Industries, Neuss, Germany
GFAP	Cow spinal cord GFAP	Rabbit	Z0334	1:2,000	Dako, Glostrup, Denmark
GLAST	C-terminus of EAAT1 of human origin	Goat	SC-7757	1:100	Santa cruz Biotechnology, Inc. Germany
GLT-1	Carboxy-terminus of rat GLT-1	Guinea pig	AB1783	1:200	Millipore, Billerica, MA, USA
mGluR1α	Mouse mGluR1a, 945–11277aa	Rabbit	G046-mGluR1a-AG	1:1,000	Frontier Institute, Japan

### Immunohistochemistry

Under deep ketamine hydrochloride anesthesia (0.12 mg/g injected i.p.—intraperitoneal), mice (*n* = 15) were transcardially perfused with a 0.9% saline solution, followed by a solution of 4% paraformaldehyde (PFA) in 0.1 M phosphate buffer (PB; pH 7.3). The brain of the mice was removed and incubated overnight in 30% sucrose in PB, and coronal sections (40 μm) were then obtained on a sliding microtome. The brain tissue from each genotype was processed simultaneously. The sections were rinsed in Tris-buffered saline (TBS, pH 7.4) containing 0.2% Triton X-100 (Tx) and incubated overnight (with agitation) at 4°C with a primary antibody raised against IBA1, GFAP, GLT1, GLAST or mGluR1α. After several rinses in TBS-Tx 0.2%, antibody binding was detected over 2 h with the corresponding biotinylated secondary antibodies (1:200; Vector Laboratories, Burlingame, CA, USA), and the antigen-antibody complex was visualized by incubating for 1 h in ABC reagent (PK-6100, vector laboratories, Burlingame, CA, USA) at room temperature and performing diaminobenzidine histochemistry. Finally, the sections were mounted onto gelatin-coated slides, air-dried and coverslipped using Cytoseal (Stephens Scientific, Camden, NJ, USA) for light microscopy analysis. Control experiments were performed by omitting either the primary or secondary antibody, or the ABC reagent, resulting in no staining.

### Double Immunofluorescence Labeling

An alternate set of brain sections from these same animals was rinsed several times in PBS-Tx 0.2% and incubated overnight in the corresponding cocktail of primary antibodies. Sections were double-labeled with a solution containing primary antibodies against IBA1and NeuN, GFAP and NeuN, GLAST and calretinin (CR), or mGluR1α and NeuN, and single-labeled with GLT1 (Table 1). After four 15 min rinses in TBS-Tx (0.2%), the secondary antibodies (1:200) were applied for 2 h at room temperature: donkey anti-goat conjugated to Alexa 488 and donkey anti-rabbit conjugated to Alexa 594 for GLAST and CR; goat anti-rabbit conjugated to Alexa 488 and goat anti-guinea pig conjugated to Alexa 594 for IBA1/NeuN, GFAP/NeuN and mGluR1α/NeuN; goat anti-guinea pig conjugated to Alexa 594 for GLT1 (Molecular Probes, Eugene, OR, USA). These sections were then counterstained with DAPI (4′,6-diamidino-2-phenylindole; Molecular Probes, Eugene, OR, USA) and mounted.

### Morphometric Analysis of Microglia Branching: Skeleton Analysis

**Figure 1:**
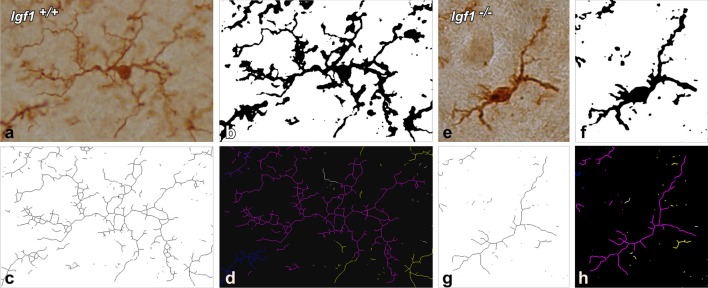
Morphometric analysis of microglial branching. For the skeleton analysis, digital images of IBA1 immunostained cells were captured with a 40× objective **(A,E)**, converted into binary images **(B,F)** and then skeletonized **(C,G)**. To evaluate microglial morphology parameters, skeletonized images were processed using the Analyze Skeleton plugin for Image J. Each glial cell is identified with a different color. The representative glia is shown in purple, and part of the skeleton of the other glial cells is shown in yellow, blue and white **(D,H)**.

To evaluate the possible modifications in the ramification of microglia in the cochlear nuclei due to IGF-1 deficiency, a skeleton analysis was performed using NIH ImageJ software (Schneider et al., 2012). The analysis of each nucleus was done on four sections from three mice per genotype. Images were captured with a 40× objective, converted into 16-bit scale and by using the thresholding function, the immunostained microglia were counted using a cell counter plug-in. The 16-bit scale images were then transformed into binary images that were subsequently, skeletonized (Figure 1). The Analyze Skeleton plug-in (version 3.1.3^2^, Arganda-Carreras et al., 2010), was used to calculate the endpoints and the processes length in each field. The obtained values were normalized and expressed as endpoints/cell and process length/cell (Morrison and Filosa, 2013; Turlejski et al., 2016; Morrison et al., 2017). The following parameters were evaluated: *(1) the number of microglial cells per field; (2) the number of microglia process endpoints per cell; and (3) the microglia process length per cell (μm)*. The endpoints/cell give us an estimate of the number of branch points of microglia, while the process length/cell gives us the mean length of microglial branches (Morrison and Filosa, 2013; Turlejski et al., 2016; Morrison et al., 2017). It is possible, at least in some instances that, after generating the skeleton, a few loops could be detected by the software in the resultant images. For pruning these loops, the software compares the original and the resulting skeleton images and by using, in our case, the method of the lowest intensity voxel, it excludes any voxel with intensities below the threshold. In that way, any possible loop/s that could affect the measurement will be eliminated. Similar to the densitometric analysis, and to avoid bias, all measurements were performed by the same experimenter blinded to the mouse genotype.

### Evaluation and Quantification of the Immunostaining

#### Light Microscopy

The brains that were processed for light microscopy were analyzed with brightfield illumination using a Nikon Eclipse photomicroscope equipped with a 40× objective. Images were captured with a DXM 1200C digital camera attached to the microscope. This study evaluates qualitatively and quantitatively the immunostaining produced by different antibodies used to label microglia, astrocytes, astroglial GLTs and mGluRs in the mouse cochlear nuclei. The qualitative parameters used to assess the immunostaining were: (1) *cell morphology* (ramified vs. bipolar), *staining intensity* (weak, moderate, or strong) and *immunopositive profiles (cell/puncta) area*. The quantitative analysis was provided through the use of custom-made macros that were written in a Pascal-like language (Xu et al., 2000; Alvarado et al., 2004). Captured color images of each selected field were digitized and the ensuing 8-bit images had a grayscale of pixel intensities that ranged from 0 (white) to 255 (black). Three animals of each genotype were used to evaluate the immunostaining in the DCN, PVCN and AVCN. In each coronal section, three microscopic fields (36.85 × 10^3^ μm^2^; dorsal, middle and ventral) were sampled 160 μm apart using a 40× objective and a total of four sections per mouse were analyzed. To quantify the immunostaining, images containing grayscale values from 0 to 255 were normalized, and then, an automatic threshold was set as two standard deviations above the value of the field. Profiles exceeding the threshold for detection were considered as labeled. *The* *mean gray level of the immunostaining* and *the immunostained area* were used as indicators of the protein levels and cell/puncta immunostained area, respectively (Fuentes-Santamaria et al., 2005; Alvarado et al., 2007b, 2014; Adams et al., 2008). The* immunostained area* gave us an estimate of the area occupied by stained profiles and it was calculated as the sum of all the immunostained cellular elements present in the field (Benson et al., 1997; Fuentes-Santamaría et al., 2012, 2013). To avoid bias, all measurements were performed by the same experimenter blinded to the animal conditions.

#### Confocal Microscopy

The brains from each genotype were processed simultaneously and the cochlear nuclei photographed under similar conditions. For appropriate image acquisition and interpretation, the following parameters were controlled: laser intensity, detector gain, pinhole aperture, number of optical sections and Z-stacking. For each dye, optical sections every 2.5 μm through the thickness of each nucleus were captured sequentially with 40× or 63× Plan Apo oil-immersion objectives, merged and saved as TIFF files using the ZEN 2009 Light Edition software. Fluorescent sections were examined with a laser scanning confocal microscope (LSM 710: Zeiss, Germany) with excitation laser lines at 405, 488 and 594 nm.

### Image Processing and Statistical Analysis

Photoshop CS3 (Adobe) and Canvas X (Deneba) were used to adjust the size, brightness and contrast of the images. The immunohistochemical and qPCR data were expressed as the means ± SD, and the statistical comparisons among genotypes were made using a one-factor analysis of variance and Scheffé’s *post hoc* analysis as necessary. A *p*-value < 0.05 was considered statistically significant, and the significance levels (α) and power (β) were set to 0.05 and 95%, respectively. Significant differences among animal groups are indicated by asterisks: *p < 0.05; **p < 0.01; and ***p < 0.001.

## Results

### Altered Microglial Morphology and IBA1 Downregulation in *Igf1^−/−^* Mice

**Figure 2:**
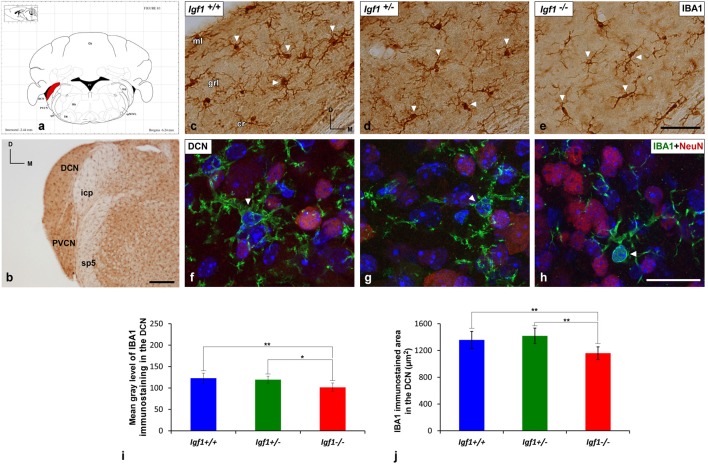
IBA1 immunostaining in the DCN of* Igf1*^−/−^ mice. **(A)** Coronal section of a wild-type mouse brain taken from the Franklin and Paxinos (2013) mouse brain atlas, to show the location of the DCN (red) and PVCN. **(B)** Representative coronal section of the cochlear nuclei in the wild-type genotype immunostained with IBA1. In *Igf1*^−/−^ mice, cells with multipolar or bipolar morphology were stained more weakly, and they had shorter processes than in *Igf1*^+/+^ and *Igf1*^+/−^ mice (arrows in** C–E**). The maximum intensity projections of confocal images of the DCN illustrate a reduction in microglial branching in *Igf1*^−/−^ mice (arrow in **H**) when compared to the other genotypes (arrows in **F,G**). Quantification of the immunostaining showed the significant decrease in the mean gray levels **(I)** and the immunostained areas **(J)** in the *Igf1*^−/−^ mouse when compared to the other genotypes. Arrows point to IBA1 immunostained cells. Cell nuclei in **(F–H)** are stained with DAPI (blue). The error bars indicate the standard deviations of the mean. Statistically significant differences among the animal groups were evaluated by one-factor analyses of variance (ANOVA; *p < 0.05; **p < 0.01). Abbreviations: Cb, Cerebellum; cr, central region of the dorsal cochlear nucleus (DCN); grl, granule/fusiform layer; IBA1, ionized-calcium-binding adaptor; icp, inferior cerebellar peduncle; IRt, intermediate reticular nucleus; ml, molecular layer; NeuN, neuronal marker; PVCN, posteroventral cochlear nucleus; sp5, spinal trigeminal nucleus; sp5OVL, spinal trigeminal nucleus, oral part, ventrolateral division; 4V, 4th ventricle; 7N, facial nucleus. Scale bars: 250 μm in **(B)**; 50 μm in (**C**; it also applies to **C,D**); 20 μm in (**H**; it also applies to **F,G**).

**Figure 3:**
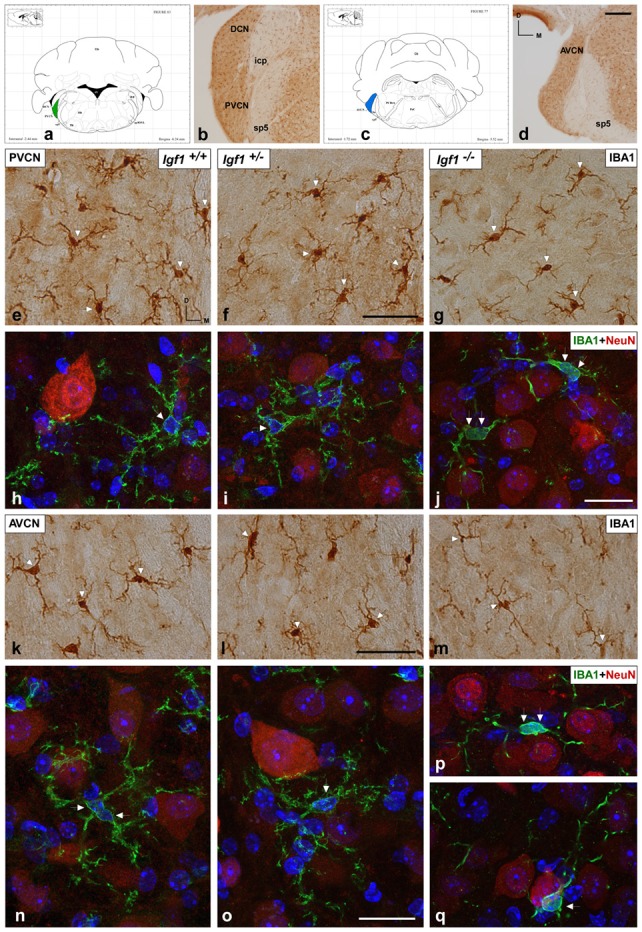
IBA1 immunostaining in the PVCN and AVCN of* Igf1*^−/−^ mice. **(A,C)** Coronal sections of a wild-type mouse brain, taken from Franklin and Paxinos (2013) mouse brain atlas, showing the location of the PVCN (green) and AVCN (blue). **(B,D)** Representative coronal sections of the ventral cochlear nucleus in the wild-type genotype immunostained with IBA1. In the PVCN and AVCN of *Igf1*^−/−^ mice, there was less staining (**G,J,M,P,Q**) relative to the *Igf1*^+/+^ **(E,H,K,N)** and *Igf1*^+/−^ mice (**F,I,L,O**). Maximum intensity projections of confocal images from the PVCN and AVCN show the reduced microglial arborization in *Igf1*^−/−^ mice as compared to the other genotypes (**H–J,N–Q** for the PVCN and AVCN; respectively). Arrows point to IBA1 immunostained cells. Cell nuclei are stained with DAPI (blue). Abbreviations: AVCN, anteroventral cochlear nucleus; Cb, Cerebellum; IBA1, ionized-calcium-binding adaptor; icp, inferior cerebellar peduncle; IRt, intermediate reticular nucleus; NeuN, neuronal marker; PnC, pontine reticular nucleus, caudal part; PVCN, posteroventral cochlear nucleus; sp5, spinal trigeminal nucleus; sp5OVL, spinal trigeminal nucleus, oral part, ventrolateral division; 4V, 4th ventricle; 7n, facial nerve; 7N, facial nucleus; 8n, vestibulocochlear nerve. Scale bars: 250 μm in (**D**; it also applies to **B**); 50 μm in (**F,J**; it also applies to **E,G,H,I**); 20 μm in (**L,O**; it also applies to **K,M,N,P,Q**).

**Figure 4:**
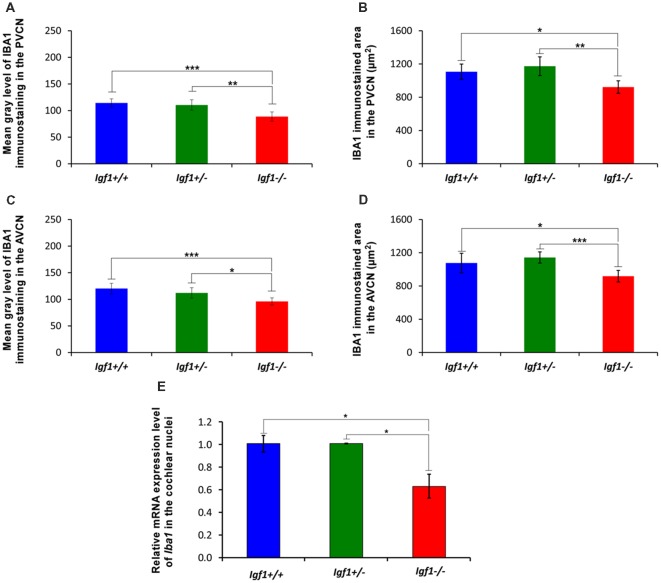
Insulin-like growth factor 1 (IGF-1) deficiency leads to *Iba1* downregulation in *Igf1*^−/−^ mice. Bar graphs showing decreases in the mean gray levels **(A,B**) and immunostained areas **(C,D)** in the *Igf1*^−/−^ mouse cochlear nuclei when compared to *Igf1*^+/+^ and *Igf1*^+/−^ mice. Similar reductions in *Iba1* expression were also detected by RT-quantitative PCR (qPCR; **E**). Error bars indicate the standard deviations of the mean. Statistically significant differences among the animal groups were evaluated by one-factor ANOVA (*p < 0.05; **p < 0.01, ***p < 0.001). Abbreviations: AVCN, anteroventral cochlear nucleus; PVCN, posteroventral cochlear nucleus.

As alterations to microglia may lead to neuronal and synaptic dysfunction (Kettenmann et al., 2013; Wu et al., 2015), the morphology of these non-neuronal cells and their IBA1 expression was studied in the cochlear nuclei of *Igf1^−/−^* mice and compared to heterozygous and wild-type mice. Regardless of the genotype, microglia in both cochlear nucleus subdivisions had a resting phenotype, characterized by a small round cell body with ramified processes (Figures 2, 3). Qualitative observations showed that, when compared to the other genotypes, *Igf1*^−/−^ mice exhibited more lightly stained microglia, which was coupled to an apparent decrease in both cell number and microglia ramification in all the nuclei analyzed (arrows in Figures 2C–E, 3E–G,K–M). To further assess the branching of microglia, reconstructed Z-stack confocal images of IBA1 stained microglia were also evaluated for each nucleus and genotype (arrows in Figures 2F–H, 3H–J,N–Q). This analysis of the confocal images also demonstrated that microglia in *Igf1*^−/−^ mice had shorter processes, which covered a smaller surface area than in *Igf1*^+/+^ and *Igf1*^+/−^ mice. These observations were corroborated by analyses of variance (ANOVA), which demonstrated a significant effect of IGF-1 deficiency over the mean gray levels of IBA1 immunostaining in the DCN (*F*_(2,22)_ = 10.35, *p* < 0.001), PVCN (*F*_(2,15)_ = 13.86, *p* < 0.001) and AVCN (*F*_(2,18)_ = 13.81, *p* < 0.001), as well as over the immunostained areas in the DCN (*F*_(2,22)_ = 10.77, *p* < 0.001), PVCN (*F*_(2,15)_ = 9.94, *p* < 0.01) and AVCN (*F*_(2,18)_ = 12.15, *p* < 0.001). Further analysis using a Scheffé’s *post hoc* test determined that the mean gray levels in the *Igf1*^−/−^ mice were significantly lower than in the *Igf1*^+/+^ (*p* < 0.01 for the DCN, *p* < 0.001 for the PVCN and AVCN) and *Igf1*^+/−^ (*p* < 0.05 for the DCN and AVCN, *p* < 0.01 for the PVCN) mice (Figures 2I, 4). Similar decreases were observed for the immunostained areas relative to the *Igf1*^+/+^ (*p* < 0.01 for the DCN, *p* < 0.05 for the PVCN and AVCN) and *Igf1*^+/−^ (*p* < 0.01 for the DCN and PVCN, *p* < 0.001 for the AVCN) mice (Figures 2J, 4). Moreover, a one-way ANOVA analysis also identified an IGF-1 dependent effect on microglial ramification. Accordingly, the morphometric analysis of microglial branching revealed statistically significant decreases in the number of microglia/field in the DCN, PVCN and AVCN of *Igf1*^−/−^ mice compared to *Igf1*^+/+^ and *Igf1*^+/−^ mice, as well as in the microglia process endpoints/cell and microglia process length/cell (Tables s 2, 3; also see 1). In accordance with the immunohistochemical data, qPCR gene expression studies detected weaker *Iba1* mRNA expression levels in the cochlear nuclei of *Igf1*^−/−^ mice relative to the other genotypes (*p* < 0.05, Figure 4E).

**Table 2:** Morphometric analysis of microglial ramification in the dorsal cochlear nucleus (DCN).

	DCN		
Genotype	Microglia/field	Microglia process endpoints/cell	Microglia process length/cell (μm)
*Igf1^+/+^* (1)	7.75 ± 0.48	45.00 ± 1.09	47.48 ± 1.08
*Igf1*^+/−^ (2)	7.25 ± 0.25	46.23 ± 2.19	49.36 ± 2.79
*Igf1*^−/−^ (3)	5.50 ± 0.29	38.22 ± 0.49	38.58 ± 1.69
*F*_(2,9)_ =	11.67 (**)	8.95 (**)	8.43 (**)
**Statistical comparison**		**Significance levels**	
1 vs. 2	NS	NS	NS
1 vs. 3	**	*	*
2 vs. 3	*	**	**

Values are means ± standard errors. Statistically significant differences among the animal groups were evaluated by one-factor analyses of variance (ANOVA; *p < 0.05; ** *p* < 0.01; NS, No significant).

**Table 3:** Morphometric analysis of microglial ramification in the posteroventral cochlear nucleus (PVCN) and anteroventral cochlear nucleus (AVCN).

	PVCN		
Genotype	Microglia/field	Microglia process endpoints/cell	Microglia process length/cell (μm)
*Igf1^+/+^* (1)	10.00 ± 0.41	52.36 ± 3.00	54.00 ± 3.98
*Igf1*^+/−^ (2)	10.75 ± 0.63	46.94 ± 2.29	52.92 ± 1.79
*Igf1*^−/−^ (3)	8.00 ± 0.41	40.67 ± 1.92	38.95 ± 2.85
*F*_(2,9)_ =	8.31 (**)	4.12 (*)	4.94 (*)
**Statistical comparison**		**Significance levels**
1 vs. 2	NS	NS	NS
1 vs. 3	*	*	*
2 vs. 3	**	*	*
		**AVCN**	
**Genotype**	**Microglia/field**	**Microglia process endpoints/cell**	**Microglia process length/cell (μm)**
*Igf1^+/+^* (1)	10.75 ± 0.63	46.80 ± 1.13	49.38 ± 1.12
*Igf1*^+/−^ (2)	10.50 ± 0.87	48.54 ± 2.30	51.83 ± 2.93
*Igf1*^−/−^ (3)	7.50 ± 0.29	40.51 ± 0.52	40.49 ± 1.79
*F*_(2,9)_ =	7.98 (**)	7.81 (**)	7.57 (**)
**Statistical comparison**		**Significance levels**	
1 vs. 2	NS	NS	NS
1 vs. 3	**	*	*
2 vs. 3	*	**	**

Values are means ± standard errors. Statistically significant differences among the animal groups were evaluated by one-factor ANOVA (*p < 0.05; **p < 0.01; NS, No significant).

### Downregulation of GFAP Expression in *Igf1*^−/−^ Mice

**Figure 5:**
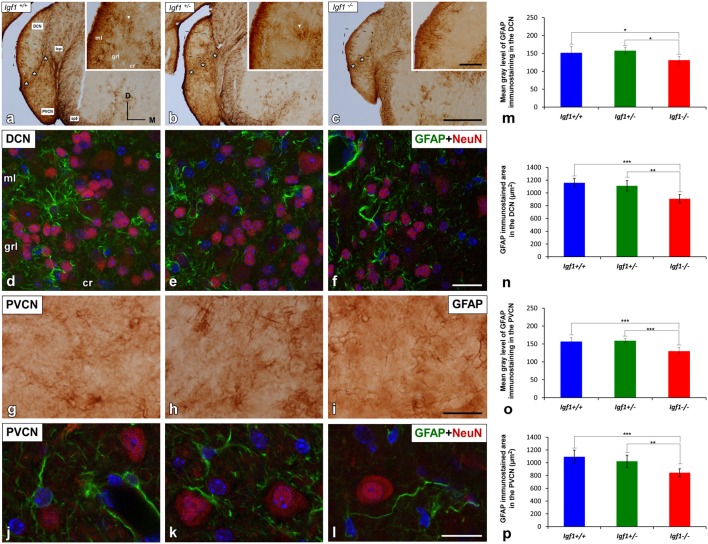
GFAP immunostaining in the DCN and PVCN of *Igf1*^−/−^ mice. GFAP immunostaining was distributed throughout the different DCN layers in all the genotypes analyzed **(A–F)**. GFAP-immunostained astrocytes were strongly stained in the molecular layer while only a few astrocytes were observed in the intermediate layer and central region of the DCN **(A–C)**. Note that some of these astrocytes group together forming patches of immunostaining (arrows in** A,B**). In the *Igf1*^−/−^ mice, GFAP immunostaining was apparently weaker in the deeper layers (grl and cr) of the DCN and in the PVCN than in *Igf1*^+/+^ and *Igf1*^+/−^ mice **(A–C,G–I)**. The spatial relationship between neurons and astrocytes in all genotypes is shown in Z-stack confocal microscopy images of the DCN **(D–F)** and PVCN **(J–L)**. Quantification of the mean gray levels **(M,O)** and stained areas **(N,P)** in these nuclei corroborated the decreases in the immunostaining. Asterisks in **(A–C)** indicate GFAP immunostaining in the granule cell domain. Statistically significant differences among the mouse genotypes were evaluated by one-factor ANOVA (*p < 0.05, **p < 0.01, ***p < 0.001). Cell nuclei in **(D–F)** and **(J–L)** are stained with DAPI (blue). The square boxes in **(A–C)** indicate the location of the higher magnification images shown in **(A–C**; insets). Abbreviations: cr, central region of the dorsal cochlear nucleus (DCN); GFAP, glial fibrillary acidic protein; grl, granule/fusiform layer; ml, molecular layer; NeuN, neuronal marker; PVCN, posteroventral cochlear nucleus; sp5, spinal trigeminal nucleus. Scale bars: 500 μm in (**C**; it also applies to **A,B**); 100 μm in the inset in (**C**; it also applies to insets in **A,B**); 20 μm in (**F,I**; it also applies to **D,E,G,H**) and 50 μm in (**L**; it also applies to **J,K**).

**Figure 6:**
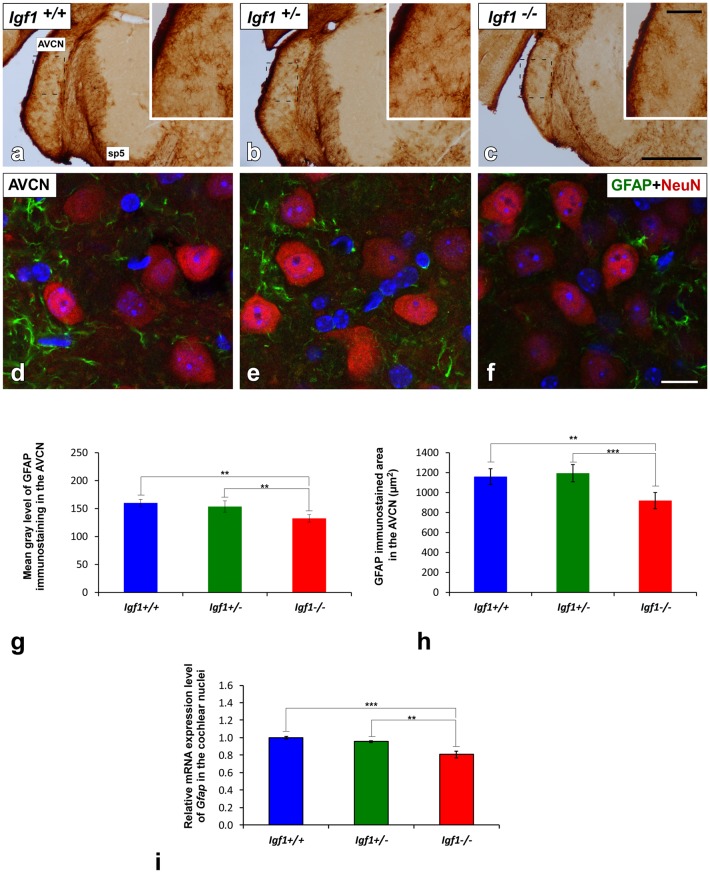
GFAP immunostaining in the AVCN of *Igf1*^−/−^ mice. Analysis of GFAP immunostaining in the *Igf1*^−/−^ mice showed that there were fewer astrocytes and they were less intensely stained **(C,F)** when compared to *Igf1*^+/+^ **(A,D)** and *Igf1*^+/−^ mice **(B,E)**. Z-stack confocal microscopy images of GFAP and NeuN double-labeling are shown for all genotypes **(D–F)**. The apparent decrease in GFAP immunostaining in the *Igf1*^−/−^ mouse was corroborated by quantifying the mean gray levels **(G)** and the stained areas **(H)**. The qualitative and quantitative data were confirmed when RT-qPCR data showed decreased *Gfap* expression in the *Igf1*^−/−^ mouse cochlear nuclei **(I)**. The error bars indicate the standard deviations of the mean. Statistically significant differences among the animal groups were evaluated by one-factor ANOVA (**p < 0.01, ***p < 0.001). Cell nuclei are stained with DAPI (blue). The square boxes in a-c indicate the location of the higher magnification images shown in (**A–C**; insets). Abbreviations: AVCN, anteroventral cochlear nucleus; GFAP, glial fibrillary acidic protein; NeuN, neuronal marker; sp5, spinal trigeminal nucleus. Scale bars: 500 μm in (**C**; it also applies to **A,B**); 100 μm in the inset in (**C**; it also applies to insets in **A,B**) and 20 μm in (**F**; it also applies to **D,E**).

Excitatory synaptic neurotransmission appears to be altered in the cochlear nucleus of *Igf1^−/−^* mice (Fuentes-Santamaría et al., 2016). Since astrocytes are modulators of mature and functional synapses, and they are crucial for the maintenance of synapses (Chung et al., 2006), the astrocytic expression of GFAP in the cochlear nuclei was examined. In all genotypes evaluated, GFAP protein was located in the cytoplasm and processes of astrocytes that were highly ramified (Figures 5, 6). Specifically, in the granule cell domain, immunostained astrocytes were densely packed and intensely stained (asterisks in Figures 5A–C). In agreement with previous observations in rodents (Burette et al., 1998; Insausti et al., 1999), GFAP immunostaining in the DCN of all genotypes, was distributed in a dorso-ventral gradient, whereby the molecular layer was more intensely stained than the intermediate granule/fusiform layer and the central region of the DCN (insets in Figures 5A–C) where astrocytes organized into patches characterized by multiple cells (arrows in Figures 5A,B). In all of the mice analyzed, irrespective of the genotype, astrocytes were associated with neurons in the cochlear nucleus, as well as with other astrocytes (Figures 5D–F). In IGF-1 deficient mouse, the immunostaining in the intermediate and deep layers of the DCN, but not in the superficial layer (ml), seemed to be weaker than in the other animal groups (Figures 5A–F). Similarly, a reduction in the immunostaining was also detected in the PVCN and AVCN of *Igf1*^−/−^ mice (Figures 5G–L, 6A–F). These decreases were confirmed by ANOVA, which showed that there was a significant effect of the IGF-1 deficiency on the mean gray levels of GFAP immunostaining in the DCN (*F*_(2,13)_ = 5.32, *p* < 0.05), PVCN (*F*_(2,18)_ = 17.32, *p* < 0.001) and AVCN (*F*_(2,12)_ = 14.99, *p* < 0.001), and on the immunostained areas in the DCN (*F*_(2,13)_ = 17.88, *p* < 0.001), PVCN (*F*_(2,18)_ = 13.37, *p* < 0.001) and AVCN (*F*_(2,12)_ = 18.33, *p* < 0.001; Figures 5M–P, 6G,H). Similarly, there was significantly less *Gfap* mRNA in the *Igf1*^−/−^ mice than in the *Igf1*^+/+^ and *Igf1*^+/−^ mice (*p* < 0.01, Figure 6I).

### IGF-1 Dependent Alterations to Astrocyte Glutamate Transporters

To determine possible abnormalities in astrocyte GLTs that might contribute to abnormal excitatory synaptic activity in the cochlear nucleus circuits of *Igf1*^−/−^ mice, as reported previously (Fuentes-Santamaría et al., 2016), the expression of GLT1 and GLAST was investigated.

#### Upregulation of EAAT2/GLT1 Expression in *Igf1^−/−^* Mice

**Figure 7:**
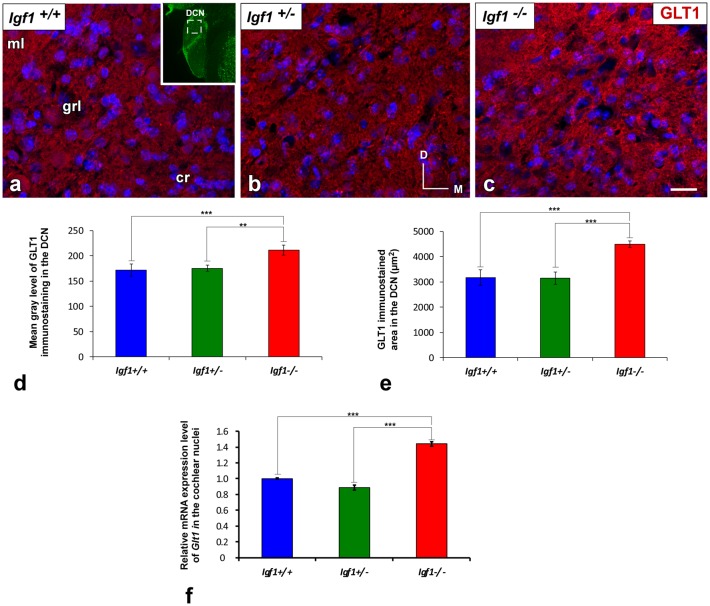
Upregulation of GLT1 in the DCN of *Igf1*^−/−^ mice. In the *Igf1*^−/−^ mouse, the strongly GLT1 immunostained puncta (red) were found in all layers of the nucleus and presumptively occupied a larger area **(C)** than in the other genotypes **(A,B)**. These qualitative observations were corroborated by significant increases in the mean gray levels and the immunostained areas **(D,E)**. Similar increases in the IGF-1 deficient DCN were also detected by quantifying *Glt1* mRNA expression **(F)**. The inset in (**A**; pseudo-colored green for higher contrast) indicates the approximate location of the fields shown in **(A–C)**. Error bars indicate the standard deviations of the mean. Statistically significant differences among the animal groups were evaluated by one-factor ANOVA (**p < 0.01, ***p < 0.001). Cell nuclei are stained with DAPI (blue). Abbreviations: cr, central region of the dorsal cochlear nucleus (DCN); GLT1, glutamate transporter 1; grl, granule/fusiform layer; ml, molecular layer. Scale bar: 20 μm in (**C**; it also applies to **A,B**).

**Figure 8:**
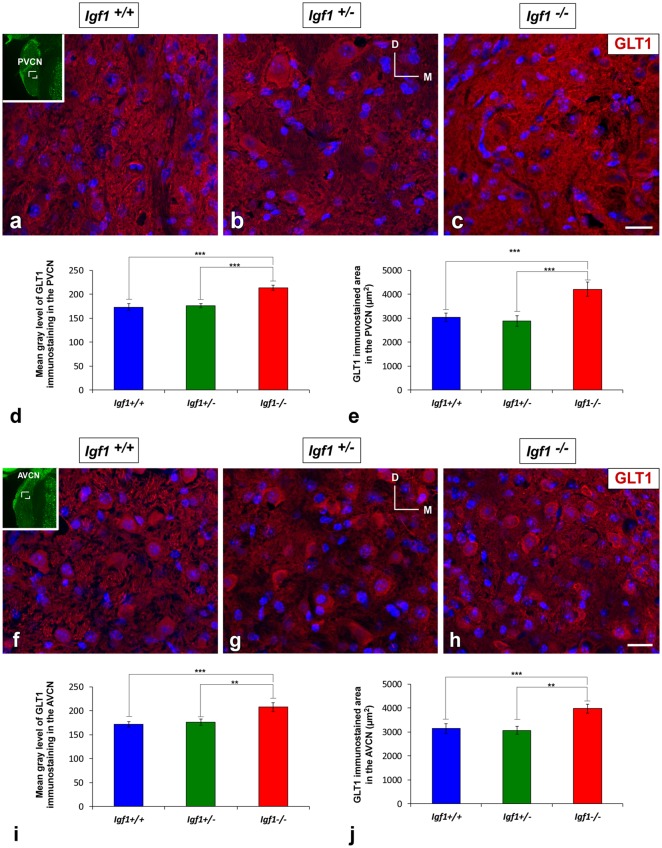
Upregulation of GLT1 in the ventral cochlear nuclei of *Igf1*^−/−^ mice. Z-stack confocal microscopy images show enhanced GLT1 immunostaining (red) in the IGF-1 deficient ventral cochlear nucleus **(C,H)** relative to the *Igf1*^+/+^ **(A,F)** and *Igf1*^+/−^ mice **(B,G)**. Quantification of the immunostaining confirmed this upregulation in both the PVCN **(D,E)** and AVCN **(I,J)**. The inset in (**A,F**; pseudo-colored green for higher contrast) indicates the approximate location of the fields shown in **(A–C,F–H)**; for the PVCN and AVCN, respectively. The error bars indicate the standard deviations of the mean. Statistically significant differences among the animal groups were evaluated by one-factor ANOVA (**p < 0.01, ***p < 0.001). Cell nuclei are stained with DAPI (blue). Abbreviations: AVCN, anteroventral cochlear nucleus; GLT1, glutamate transporter 1; PVCN, posteroventral cochlear nucleus. Scale bar: 20 μm in (**C,H**; it also applies to **A,B,F,G**).

Regardless of the genotype, GLT1 immunostaining was evident as dense punctate labeling that was similarly distributed throughout the cochlear nuclei (Figures 7, 8). In the DCN, the GLT1 stained puncta were distributed throughout the molecular layer, granule/fusiform layer and the central region of the DCN (Figures 7A–C). Similar diffuse punctate neuropil staining was also observed in the PVCN (Figures 8A–C) and AVCN (Figures 8F–H). Although the distribution of GLT1 immunostaining was similar in the different genotypes, the GLT1 puncta in *Igf1^−/−^* mice were apparently more strongly stained and they seemed to occupy a larger extension in both subdivisions of the cochlear nucleus (Figures 7, 8). These qualitative immunohistochemical appreciations were confirmed by ANOVA, which showed a significant effect of the absence of IGF-1 on the mean gray level of GLT1 in the DCN (*F*_(2,10)_ = 15.94, *p* < 0.001), PVCN (*F*_(2,10)_ = 61.48, *p* < 0.001) and AVCN (*F*_(2,9)_ = 32.92, *p* < 0.001) and also on the immunostained areas in the DCN (*F*_(2,10)_ = 28.16, *p* < 0.001), PVCN (*F*_(2,10)_ = 41.85, *p* < 0.001) and AVCN (*F*_(2,9)_ = 28.66, *p* < 0.001). As demonstrated using Scheffé’s *post hoc* test, the mean gray levels in the *Igf1*^−/−^ mice were significantly higher than in the *Igf1*^+/+^ (*p* < 0.001 for DCN, PVCN and AVCN) and *Igf1*^+/−^ (*p* < 0.01 for DCN and AVCN, *p* < 0.001 for PVCN) mice (Figures 7D, 8D,I). Likewise, the GLT1 immunostained areas were also larger in the *Igf1*^−/−^ mice than in the *Igf1*^+/+^ (*p* < 0.001 for all nuclei) and *Igf1*^+/−^ (*p* < 0.001 for DCN and PVCN; *p* < 0.01 for AVCN) mice (Figures 7E, 8E,J). Corroborating the protein expression data, *Glt1* mRNA expression in the *Igf1*^−/−^ mice cochlear nuclei was significantly stronger than in the other genotypes (*p* < 0.001; Figure 7F).

#### GLAST Expression in *Igf1^−/−^* Mice

**Figure 9:**
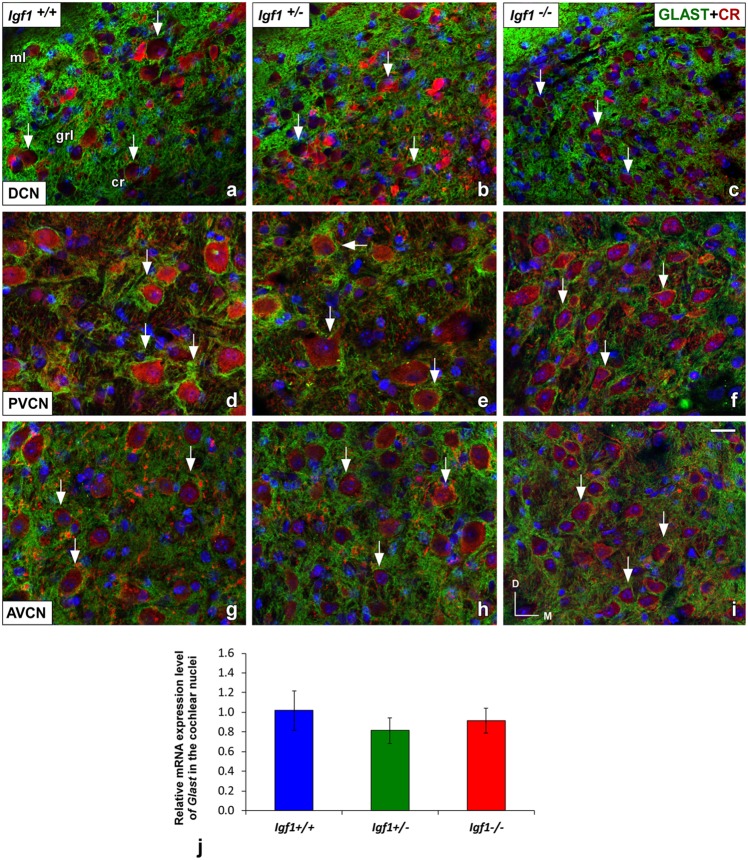
Glutamate aspartate transporter (GLAST) immunostaining in the *Igf1*^−/−^ mice cochlear nuclei. In all genotypes, GLAST stained puncta (green) were densely distributed throughout the neuropil and on the soma of DCN **(A–C)**, PVCN **(D–F)** and AVCN **(G–I)** neurons immunostained with CR (red). No differences were observed in the distribution of the immunostaining among the three groups. The analysis of *Glast* expression levels by RT-qPCR confirmed these immunohistochemical results **(J)**. The error bars indicate the standard deviations of the mean. Arrows point to CR-immunostained cells. Cell nuclei are stained with DAPI (blue). Abbreviations: AVCN, anteroventral cochlear nucleus; cr, central region of the DCN (dorsal cochlear nucleus); CR, calretinin; GLAST, glutamate aspartate transporter; grl, granule/fusiform layer; ml, molecular layer; PVCN, posteroventral cochlear nucleus. Scale bar: 20 μm in (**I**; it also applies to **A–H**).

**Figure 10:**
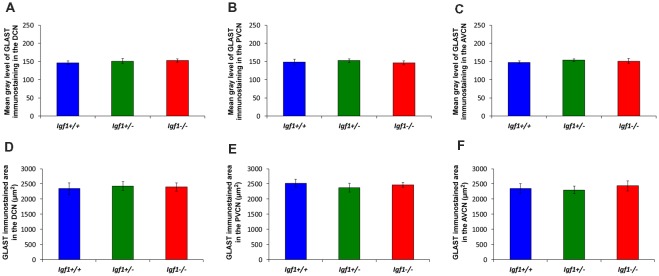
Histograms showing the mean gray levels of GLAST immunostaining and the immunostained areas in the cochlear nuclei of *Igf1*^−/−^, *Igf1*^+/−^ and *Igf1*^+/+^ mice. Analysis of the immunostaining indicated that there were no significant differences among the genotypes in either the mean gray levels of immunostaining **(A–C)** or in the immunostained areas **(D–F)** in the DCN, PVCN and AVCN. The error bars indicate the standard deviations of the mean. Abbreviations: DCN, dorsal cochlear nucleus; PVCN, posteroventral cochlear nucleus; AVCN, anteroventral cochlear nucleus.

Like GLT1, a dense network of GLAST immunostained puncta was evident in the neuropil and around the soma of DCN, PVCN and AVCN neurons in *Igf1^+/+^, Igf1^+/–^* and *Igf1*^−/−^ mice (Figure 9). However, in contrast to GLT1 there appeared to be no significant differences in the immunostaining among the distinct mouse genotypes, in either the mean gray levels of GLAST immunostaining in the DCN (*F*_(2,7)_ = 0.94, NS), PVCN (*F*_(2,9)_ = 1.01, NS) and AVCN (*F*_(2,11)_ = 1.56, NS) or in the immunostained areas in the DCN (*F*_(2,7)_ = 0.24, NS), PVCN (*F*_(2,9)_ = 1.56, NS) and AVCN (*F*_(2,11)_ = 0.93, NS; Figure 10). In accordance with these immunohistochemical findings, there were also no differences in *Glast* gene expression in the cochlear nuclei of *Igf1*^−/−^ mice relative to the *Igf1*^+/−^ and *Igf1*^−/−^ mice (Figure 9J).

### Downregulation of mGluRs in *Igf1^−/−^* Mice

**Figure 11:**
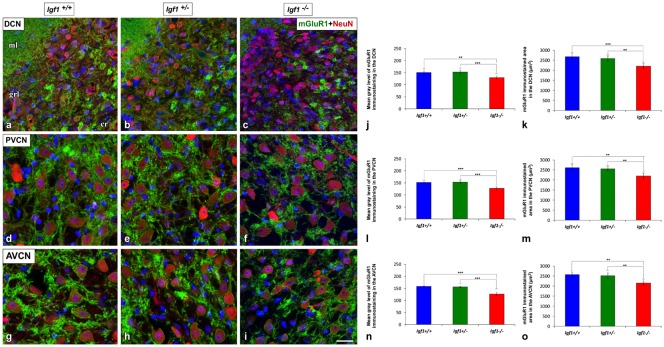
Downregulation of mGluR1α in the cochlear nuclei of* Igf1*^−/−^ mice. In the *Igf1*^−/−^ mice, there was less mGluR1α staining (green) in the molecular and granule layers of the DCN than in the other genotypes **(A–C)**. Similar decreases were also detected in *Igf1*^−/−^ mouse PVCN **(D–F)** and AVCN **(G–I)**. Quantification confirmed these qualitative decreases in the *Igf1*^−/−^ mouse relative to the *Igf1*^+/+^ and *Igf1*^+/−^ mice **(J–O)**. The error bars indicate the standard deviations of the mean. Statistically significant differences among the animal groups were evaluated by one-factor ANOVA (**p < 0.01, ***p < 0.001). Neurons are stained with NeuN antibody (red) and cell nuclei are stained with DAPI (blue). Abbreviations: AVCN, anteroventral cochlear nucleus; cr, central region of the dorsal cochlear nucleus (DCN); grl, granule/fusiform layer; ml, molecular layer; mGluR1, metabotropic glutamate receptor 1; NeuN, neuronal marker; PVCN, posteroventral cochlear nucleus. Scale bar: 20 μm in (**I**; it also applies to **A–H**).

**Figure 12:**
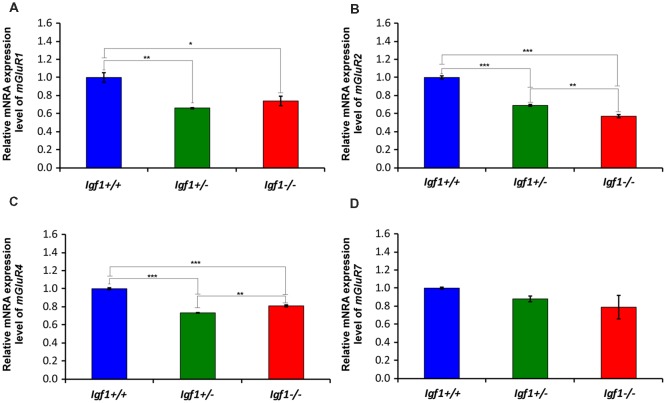
The loss of IGF-1 leads to *mGluR1α, mGluR2α, mGluR4α and mGluR7α* downregulation. The *mGluR1α, mGluR2α, mGluR4α and mGluR7α* mRNA expression was determined by RT-qPCR in 4-month-old *Igf1*^−/−^, *Igf1*^+/−^ and *Igf1*^+/+^ mice. The results demonstrate a decline in *mGluR1α* **(A)**, *mGluR2α* **(B)**, *mGluR4α* **(C)** and *mGluR7α* **(D)** expression in the *Igf1*^−/−^ mouse when compared with *Igf1*^+/−^ and *Igf1*^+/+^ mice. Note that the decrease in *mgluR7α* expression in the *Igf1*^−/−^ mice was not statistically significant. Also, they show a significant downregulation of *mGluR1α* **(A)**, *mGluR2α* **(B)** and *mGluR4α* in heterozygous mice compared with their age-matched control mice. The error bars indicate the standard deviations of the mean. Statistically significant differences among the animal groups were evaluated by one-factor ANOVA (*p < 0.05; **p < 0.01, ***p < 0.001).

As the upregulated GLT1 expression is suggestive of abnormal glutamate uptake, the mRNA and protein expression of mGluRs were also examined in the *Igf1^−/−^* mice cochlear nuclei. As mGluR1α is expressed abundantly in the cochlear nuclei (Lu, 2014), its expression was evaluated at both the mRNA and protein levels, whereas the expression of *mGluR2*, *mGluR*4 a*nd mGluR7* was only based on qPCR analyses. mGluR1α staining was detected in the DCN of *Igf1*^+/+^, *Igf1*^+/−^ and *Igf1*^−/−^ mice, mostly in the neuropil of the molecular layer and on the dendrites of fusiform and cartwheel cells in the granule/fusiform layer (Figures 11A–C), (Bilak and Morest, 1998; Petralia et al., 2000). Regardless of the genotype, the immunostaining in the PVCN and AVCN was observed in globular and stellate cells which were identified according to their location within the nucleus, as shown previously (Bilak and Morest, 1998; Figures 11D–I). In *Igf1*^−/−^ mice, the mGluR1α staining levels in the dorsal and ventral cochlear nucleus subdivisions were weaker than in the other genotypes (Figure 11). When quantified, immunostaining revealed a significant effect of the lack of IGF-1 on the mean gray level of mGluR1α in the DCN (*F*_(2,13)_ = 18.17, *p* < 0.001), PVCN (*F*_(2,14)_ = 51.59, *p* < 0.001) and AVCN (*F*_(2,9)_ = 56.55, *p* < 0.001) and also on the immunostained areas in the DCN (*F*_(2,13)_ = 18.17, *p* < 0.001), PVCN (*F*_(2,14)_ = 16.03, *p* < 0.01) and AVCN (*F*_(2,9)_ = 19.91, *p* < 0.001). According to the *post hoc* test, the mean gray levels in the *Igf1*^−/−^ mice were significantly lower than in the *Igf1*^+/+^ (*p* < 0.01 for the DCN, *p* < 0.001 for the PVCN and AVCN) and *Igf1*^+/−^ (*p* < 0.001 for all nuclei) mice (Figures 11J,L,N). Likewise, the immunostained areas of mGluR1α were also smaller in the *Igf1*^−/−^ mice than in the *Igf1*^+/+^ (*p* < 0.001 for the DCN, *p* < 0.01 for the PVCN and AVCN) and *Igf1*^+/−^ (*p* < 0.01 for all nuclei) mice (Figures 11K,M,O). Significant decreases in *mGluR1, mGluR2* and *mGluR4* gene expression were detected in the *Igf1*^−/−^ mouse cochlear nucleus relative to the wild-type mice. Although *mGluR7* expression also appeared to be weaker, this difference did not reach statistical significance (Figure 12).

## Discussion

The present study demonstrates that IGF-1 deficiency leads to alterations in glial morphology, a decreased expression and protein accumulation in IBA1 and GFAP, an upregulation of GLT1 but not GLAST, and the downregulation of groups I, II and III mGluRs. Our previous findings in *Igf1^−/−^* mice demonstrated that primary cochlear afferents upregulate their VGluT1 protein but not the vesicular GABA transporter (VGAT), suggesting that presynaptic excitatory neurotransmission may be impaired. This synaptic plasticity might be mediated by MEF2 proteins, which are downregulated in both the cochlea and the cochlear nucleus, and which may reflect enhanced synaptic efficacy (Fuentes-Santamaría et al., 2016). As an activity-dependent factor, rapid elevations of IGF-1 within neurons in response to modifications in cochlear activity have been associated with synaptic rearrangements in the rat cochlear nucleus (Alvarado et al., 2007a; Fuentes-Santamaría et al., 2007, 2012, 2013). In other brain structures like the hippocampus, IGF-1 is also a regulator of excitatory synaptic transmission (Ramsey et al., 2005; Xing et al., 2007; Deak and Sonntag, 2012). Indeed, the effects of a reduced density of glutamatergic terminals in the hippocampus of mice with low-serum IGF-1 levels is ameliorated by continued systemic IGF-1 administration (Trejo et al., 2007).

The effects of IGF-1 on glial cells have been studied in different systems. When non-astrocytic inner retinal glia-like cells are stimulated by IGF-1 in the avian retina, retinal neurons and Müller glia are more vulnerable to excitotoxic damage (Fischer et al., 2010). In the mouse retina, IGF-1 deficiency causes age-associated retinal gliosis (Arroba et al., 2018). In cerebellar cultures, blocking the IGF-1 receptor in astrocytes reduces their capacity to rescue neurons damaged by oxidative stress, suggesting that this growth factor is crucial for these non-neuronal cells to exert their protective effects (Genis et al., 2014). Moreover, transgenic mice overexpressing IGF-1 in the retina develop gliosis and microgliosis, along with impaired glutamate recycling, which leads to cell death. Hence, glial cells appear to be involved in the regulation of excitatory synaptic function (Villacampa et al., 2013). Our data demonstrate that *Igf1*^−/−^ mice have less *Iba1* mRNA and, consequently, lower IBA1 protein levels along with decreases in the mean gray levels of IBA1 immunostaining and immunostained areas in both cochlear nucleus subdivisions. *Igf1*^−/−^ mice display structural modifications of microglia including decreases in the number of cells per field, shorter processes length and a reduction in microglial branching as compared to heterozygous and wildtype mice. The contribution of microglia to the adaptive responses that take place in the auditory nuclei in response to cochlear damage induced by acoustic trauma or cochlear ablation has been well documented (Fuentes-Santamaría et al., 2012; Dinh et al., 2014; Janz and Illing, 2014; Baizer et al., 2015). In this regard, lesion-induced microglial activation may exert regulatory influences on cochlear nucleus synapses, contributing to structural and functional remodeling. Recent data from microglia depletion models indicated that the loss of microglia during brain development leads to defective glial-synapse communication and aberrant synaptic maturation (Paolicelli and Ferretti, 2017). Indeed, the absence of IGF-1 leads to an ineffective refinement of cochlear synapses during postnatal maturation, which results in dysfunctional excitatory connections in the adult cochlear nucleus (Camarero et al., 2001; Riquelme et al., 2010; Fuentes-Santamaría et al., 2016). Given that microglial motility is primarily involved in reshaping neuronal circuits during development, and in supporting and maintaining active synapses in the adult brain, limited microglial branching due to IGF-1 deficiency may produce defective neuronal-glial communication and consequently, abnormal synaptic transmission in auditory nuclei.

Astrocytes continuously exchange signals with pre- and postsynaptic elements at the tripartite synapse, and they are also crucial for the formation and maintenance of glutamatergic synapses due to their dynamic involvement in the processing and integration of synaptic information (Ricci et al., 2009; Villalba and Smith, 2011; Kim et al., 2017; Papouin et al., 2017). Our data in the *Igf1^−/−^* mouse reveal a significant decrease in GFAP protein and mRNA, as well as reductions in astrocyte density. In this regard, the loss of GFAP in *Igf1*^−/−^ mice results in increased synaptic plasticity and altered GLT activity, suggesting that GFAP expression is essential to correctly regulate glutamatergic neurotransmission (Hughes et al., 2004). It is worth noting that the morphological and functional plasticity of the astrocyte is achieved through polymerization/assembly and depolymerization/disassembling of GFAP which acts a scaffolding network for the translocation of GFAP-associated functional molecules (Wang and Parpura, 2018), such as the GLTs. Accordingly, it has been proposed that the glutamate-mediated localization of GLT1 and GLAST in the astrocyte membrane is highly dependent on the actin cytoskeleton of this glial cell (Duan et al., 1999; Zhou, 2004). Astrocyte dysfunction may modify the coverage of neurons by glia, increasing neuronal communication and influencing glutamate concentrations and therefore, the activity of carriers and receptors. GLT1 and GLAST are both high-affinity, sodium-dependent GLTs located on perisynaptic processes of astrocytes and closely associated with excitatory synapses. In cultures of chick cerebellar Bergmann glia cells, GLT1 activity is modulated by IGF-1 signaling (Gamboa and Ortega, 2002) and thus, the absence of this factor may alter excitatory signaling.

GLT1 dysfunction has been linked to several neurological disorders in which glutamate homeostasis is impaired (Verkhratsky et al., 2012; Soni et al., 2014). For instance, upregulation of GLT1 expression improves signs of Huntington’s disease in symptomatic R6/2 mice (Miller et al., 2008) and enhanced GLT1 expression also has been detected in the prefrontal cortex of schizophrenic patients, suggesting impaired glutamatergic transmission in this disease (Matute et al., 2005). GLT1 also appears to be upregulated in *Igf1^−/−^* mice, the predominant glial GLT, while the expression and accumulation of the GLAST protein were not modified. In line with these observations, GLT1 immunoreactivity is enhanced in cerebellar synaptosomal preparations of *Gfap*^−/−^ mice when compared to wild-type animals (Hughes et al., 2004). Such upregulation in cerebellar synaptic GLT1 protein expression may facilitate the rapid uptake of synaptic glutamate through the activation of mGluRs, suggesting a pivotal role for GFAP in trafficking GLTs. Based on these observations, our results provide evidence of IGF-1-dependent regulation of astrocytes, that alters glial glutamate transport capacity and therefore, excitatory synapses. Although the number of astrocytes and/or transporters per cell was not assessed for each genotype in the current study, it is plausible to postulate that an upregulation in GLT1 in the mutant mouse would contribute to clear excessive glutamate resultant from increased neuronal interactions in response to astrocytes loss and/or dysfunction. Indeed, GLT1 is responsible for most of the glutamate uptake (90%) around excitatory synapses (Lehre and Danbolt, 1998), although the mechanisms that drive the increase in GLT1 in the *Igf1*^−/−^ mice are still unclear.

GLT1 overexpression near synapses could have important consequences for the kinetics of glutamate uptake and hence, on mGluR levels in the cochlear nuclei. For instance, an upregulation in GLT1 in the *Igf1^−/−^* cochlear nuclei will reduce the amount of glutamate available in the synaptic cleft, thus resulting in decreased glutamate binding to mGluRs (Huang et al., 2004). This decrease, in turn, would lead to significantly dampen the gene expression levels of *mGluR1*, *mGluR2*, *mGluR4* and* mGluR7*, as demonstrated in this study. In accordance with our data, previous studies have demonstrated that *mGluR1* is detected in the DCN of rodents under normal physiological conditions, particularly on unipolar brush cells and cartwheel cells, and also in globular bushy cells and stellate cells in the PVCN and AVCN (Bilak and Morest, 1998; Kemmer and Vater, 2001). Although the mGluR1 in the cochlear nuclei is mostly found post-synaptically, it has also been detected in presynaptic elements (Petralia et al., 1996; Wright et al., 1996). mGluR2 is also expressed in the cochlear nuclei while the expression of mGluR4 and mGluR7 remains unclear (Lu, 2014). *In vivo* and *in vitro* studies in the chick cochlear nucleus, nucleus magnocellularis, have demonstrated that pharmacological blockade of groups I and II mGluR activation provokes neuronal degeneration, suggesting a pivotal role for these receptors in regulating neuronal survival (Nicholas and Hyson, 2004; Diaz et al., 2009; Carzoli and Hyson, 2011, 2014). The activation of these receptors also regulates glutamate uptake at the cochlear nucleus-auditory nerve synapse, preventing the excitotoxic accumulation of extracellular glutamate (Carzoli and Hyson, 2014). Whole cell recordings in brain slice preparations demonstrated that the activity of mGluRs suppresses GABAergic transmission (Lu, 2007), highlighting their role in achieving balanced excitation and inhibition in the nucleus magnocellularis.

In summary, adult *Igf1*^−/−^ mice show modifications in the morphological features of the glutamatergic synapses (presynaptic and postsynaptic components) that lead to excitatory synaptic plasticity, which involves not only auditory neurons but also glial cells. Although the role of microglia in this process is unclear, their reduced arborization may result in more prolonged microglia response times and therefore, lead to decreased modulation of neuronal activity. On the other hand, the reduction in the fine astrocytic processes may facilitate neuronal interactions that, along with an enhanced expression of GLT1, would contribute to regulation of synaptic glutamate resulting from excessive neuronal activation.

## Conclusion

The results presented here suggest that absence of IGF-1 leads to central adaptive events in the adult *Igf1^−/−^* cochlear nuclei, which include structural impairment of microglia and astrocytes, upregulation in GLT1 expression, and downregulation of mGluRs. These morphological alterations may contribute to adaptation of auditory neurons and their synaptic connections to changing levels of activity, due to the imbalances in neurotransmission. Determining the molecular, biochemical and morphological mechanisms underlying neuronal plasticity in a mouse model of hearing deficits will give us insight into new therapeutic strategies that could help to maintain or even improve residual hearing when human deafness is related to IGF-1 deficiency.

## Author Contributions

All authors had full access to all the data in the study and take responsibility for the integrity of the data and the accuracy of the data analysis. VF-S, JA and IV-N: study concept and design. VF-S, JA and LR-R: acquisition of data, statistical analysis and interpretation of data. VF-S and JA: drafting of the manuscript. VF-S, JA, LR-R, IV-N and JJ: critical revision of the manuscript for important intellectual content. IV-N, VF-S and JJ: obtaining funding.

## Conflict of Interest Statement

The authors declare that the research was conducted in the absence of any commercial or financial relationships that could be construed as a potential conflict of interest.

## Footnotes

^1^https://bioinfo.appliedbiosystems.com/genome-database/gene-expression.html

^2^https://imagej.net/AnalyzeSkeleton
